# Targeting MLKL-Driven Necroptosis: A Therapeutic Target in Inflammation and Host Defense

**DOI:** 10.3390/biom16030360

**Published:** 2026-02-28

**Authors:** Sarmistha Saha, Luciano Saso, Brigitta Buttari

**Affiliations:** 1 Department of Biotechnology, Institute of Applied Sciences & Humanities, GLA University, Mathura 281406, Uttar Pradesh, India; 2 Department of Physiology and Pharmacology “Vittorio Erspamer”, Sapienza University of Rome, 00161 Rome, Italy; luciano.saso@uniroma1.it; 3 Department of Cardiovascular, Endocrine-Metabolic Diseases and Aging, Italian National Institute of Health, 00161 Rome, Italy

**Keywords:** MLKL, necroptosis, TNFR1

## Abstract

Necroptosis is a regulated form of programmed cell death that helps the body defend itself against infections and cellular stress, especially when apoptosis is blocked. At the center of this process is mixed lineage kinase domain-like (MLKL) protein, the final effector of necroptosis, which is activated downstream of receptor-interacting protein kinase 3 (RIPK3). Once phosphorylated, MLKL changes shape, assembles into oligomers, moves to cellular membranes, and disrupts membrane integrity, ultimately causing cell death. While this RIPK3-MLKL pathway has been well described, it is becoming increasingly clear that MLKL regulation is more complex than originally thought. Recent findings show that MLKL can be modified and activated through alternative mechanisms, even in the absence of RIPK3, and that post-translational modifications such as ubiquitination further fine-tune its activity. Notably, deleting RIPK3 or MLKL does not consistently resolve inflammatory phenotypes in experimental models, suggesting that MLKL has context-dependent functions that extend beyond its role in necroptosis. In line with this idea, MLKL has been implicated in inflammatory signaling, interferon responses, and innate immunity, and is frequently targeted by viruses seeking to evade host defenses. Beyond infections, aberrant MLKL activation contributes to a wide range of chronic diseases, including atherosclerosis, cardiometabolic disorders, liver disease, neurodegeneration, and cancer. In these settings, sustained MLKL-mediated membrane damage and release of danger signals drive ongoing inflammation and tissue injury rather than protective cell elimination. In this review, we provide an overview of MLKL structure, activation, and regulation in both necroptotic and non-necroptotic contexts. We also discuss emerging therapeutic strategies aimed at targeting MLKL activation, membrane engagement, and stability, and highlight key unanswered questions that must be addressed to translate MLKL biology into effective clinical interventions.

## 1. Introduction

Programmed cell death serves as an important defense against microbial diseases. Apoptosis and autophagy represent the main forms of programmed cell death. The molecular pathways responsible for removing infected or damaged cells have been extensively studied. Although necrosis is traditionally considered unregulated, necroptosis represents a distinct form of cell death that combines features of both apoptosis and necrosis, and is often referred to as “programmed necrosis” [1]. Necroptosis is a regulated cell death pathway that eliminates infected or damaged cells when apoptosis fails and promotes immune activation. While protective, its dysregulation contributes to inflammatory and ischemic diseases, and its inhibition reduces disease severity in experimental models [2,3,4,5].

At the molecular level, the formation of the tightly regulated necrosome complex can be initiated by death receptors such as TNF receptor 1 (TNFR1), by cell-surface toll-like receptors, by the cytosolic sensor DNA activator of interferons (DAI), which may detect viral RNA, and potentially by additional upstream signals [6]. The formation of a necrosome complex is dependent on the activation of receptor-interacting protein kinase (RIPK)-1/3 and mixed-lineage kinase domain-like (MLKL). The necrosome contains several proteins such as RIPK1 and RIPK3, DED proteins, procaspase-8/-10, c-FLIP, and MLKL, which serve as scaffolding for RIPK3 clustering and activation. Active RIPK3 phosphorylates its major target, MLKL, initiating the effector phase of necroptotic cell death.

MLKL has an N-terminal four-helix bundle (4HB) domain and a C-terminal pseudokinase domain. RIPK3 phosphorylation alters MLKL’s conformation, causing it to oligomerize and release its 4HB domain [7]. This module binds to a variety of phospholipids, including phosphatidylinositol 5-phosphate (PI(5)P) and PI(4,5)P_2_, as well as cardiolipin [8]. Oligomerized MLKL binds to phospholipids in both plasma and intracellular membranes, promoting its translocation to the plasma membrane. Once at the membrane, MLKL compromises membrane integrity, leading to necrotic cell death. Importantly, inhibition of phosphatidylinositol phosphate synthesis selectively blocks TNF-α–induced necroptosis while leaving apoptosis unaffected. Recent studies have shown that RIPK3 can be activated by innate immunological triggers other than TNF-α, leading to a broader understanding of necroptosis. The cytosolic nucleic acid sensor ZBP1, also known as Z-form RNA binding protein 1 (ZBP1)/DAI, has two Za domains that detect viral nucleic acids and an RHIM domain that interacts with other proteins with the RHIM structure [9]. ZBP1 detects herpesvirus and influenza virus infections and activates RIPK3, resulting in necroptosis [10]. TLR3 and TLR4 recognize viral and microbial ligands (double-stranded RNA and lipopolysaccharide, respectively) and activate RIPK3 through the adaptor protein TRIF [11].

RIPK3-deficient mice show that this kinase plays a crucial role in vertebrate defense and that necroptosis helps eliminate pathogens in two ways. It reduces pathogen spread by destroying host cells and preventing them from becoming a pathogen factory. Secondly, it activates the host’s adaptive immune response by causing cell death and debris release [10]. Certain substances, such as nuclear and cytosolic proteins, might trigger a strong immunological response [12]. Necroptosis, while potentially beneficial for pathogen clearance, can have severe pathogenic repercussions if not activated properly [12]. This review explores how viruses control host defenses and antiviral inflammation by targeting RIPK3 and the necroptosis pathway. The review is organized into three main sections. First, the molecular mechanisms underlying target activation are examined. Next, current evidence regarding viral antagonists of necroptosis is critically analyzed. Finally, the therapeutic potential of translating this knowledge into the development of targeted inhibitors is discussed.

## 2. Structure of Mixed Lineage Kinase Domain-like (MLKL) Protein

**Figure 1:**
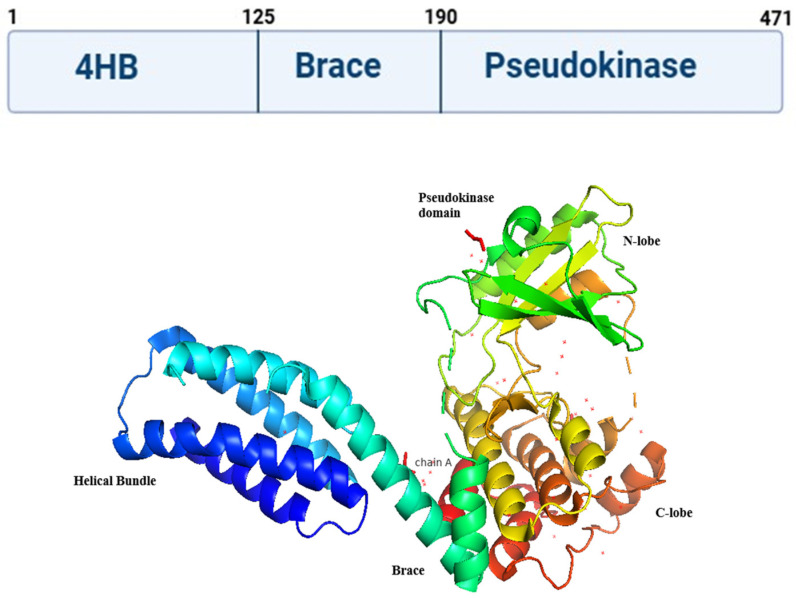
A surface representation of MLKL from crystal structures: 4BTF.

Murphy et al. [13] described the full-length crystal structure of mouse MLKL. However, determining the full-length crystal structure of human MLKL is a continuing challenge. The structure reported by Murphy et al. revealed that mouse MLKL is composed of an N-terminal four-helix bundle (4HB; residues 1–117), a two-helix linker known as the brace domain (residues 129–169), and a C-terminal pseudokinase domain (PSKD; residues 171–464) (Figure 1) [14]. MLKL is classified as a pseudokinase because its C-terminal kinase-like domain lacks two of the three key catalytic residues normally required for phosphoryl transfer in active protein kinases. By binding and inhibiting the 4HB domain’s executor function in its dormant, nonactivated state, the carboxy terminal pseudokinase domain performs a regulatory function [15,16,17,18,19]. Furthermore, the pseudokinase domain functions as a signal integrator, where RIPK3’s phosphorylation of the activation loop acts as an activation cue. It is believed that this process causes the pseudokinase to undergo a conformational shift that facilitates the exposure of the 4HB domain, allowing MLKL to oligomerize, translocate to, and permeabilize the plasma membrane [20]. A brace domain, composed of two alpha helices, links these domains [15,16]. The brace domain regulates MLKL oligomerization, membrane transport, and release of cytotoxic 4HB. Studies in humans and mice show that the 4HBD is essential for MLKL-induced necroptosis. Without necroptotic stimulation or endogenous MLKL activation, forced expression of a dimerizable 4HBD in MLKL-expressing cells induces necroptosis [17,21]. This suggests that under necroptotic stimulation, post-translational changes on MLKL create a structurally active conformation. This allows the 4HBD to become accessible and form oligo-multimeric complexes with other active MLKL molecules, executing necroptosis [8]. The PSKD can bind ATP without hydrolyzing it, making this domain virtually inert. However, this domain is required for RIPK3 binding and subsequent RIPK3-mediated MLKL phosphorylation, a process known as the molecular switch [8,13]. RIPK3-mediated phosphorylation causes MLKL to interconvert to a close yet active form [22]. This causes MLKL to be released from RIPK3, high-order MLKL oligomers to form through 4HBD interaction, and membrane destabilization, which leads to pore formation [7,8,14,17,21,22,23,24,25,26]. Human MLKL possesses a closed-active-like conformation and a possible open-inactive conformation with an analogous K230-Q356 hydrogen bond [22].

Human MLKL is linked to RIPK3 in baseline circumstances [22,27]. However, unlike mouse MLKL, alanine substitution of K230 or Q356 does not fully activate and destroy MLKL [22,28]. After a necroptosis stimulation, MLKL dissociates from RIPK3 due to RIPK3-mediated phosphorylation, which is thought to destabilize the MLKL-RIPK3 connection [22]. Human MLKL dissociates and forms a closed-active conformation with the K230 bound to an E250 via a salt bridge, resulting in an intact catalytical C-spine. This, in turn, would necessitate the presence of ATP or another metabolite ligand at the ATP-binding region in order to modulate the MLKL conformational change, switching from active to inactive and so influencing necroptotic kinetics [22,29].

In HeLa cells, which do not express RIPK3, MLKL is phosphorylated at serine 125 (S125) during prolonged mitosis, indicating that its regulation can occur independently of the canonical RIPK3-mediated necroptotic pathway. In addition, TAM family kinases (Tyro3, Axl, and Mer) phosphorylate tyrosine 376 (Y376) on MLKL, promoting its stabilization and facilitating oligomerization, which are key steps for MLKL activation and membrane targeting [30,31]. Recent phosphomimetic substitution of residues near the activation loop of human MLKL showed that the T374D mutation completely suppressed necroptotic signaling by reducing RIPK3-mediated phosphorylation of T357/S358 [22]. Both T374 and S125 are phosphorylated in a cell cycle-dependent manner. The kinases responsible for these phosphorylation events remain unidentified. The interaction between human RIPK3 and human MLKL requires the formation of stable complexes involving the RIPK3 kinase domain and the MLKL PSKD.

MLKL is found to be ubiquitinated after activation. Following these events, MLKL molecules engage with 4HBD to create oligomeric structures of various sizes (trimers and/or multimers). It has now been demonstrated that this process occurs in the cytoplasm before membrane localization. At the membrane, particular 4HBD residues bind PIPs or cardiolipin [18,19,25,29]. This allows human MLKL to move from the cytosol to the plasma membrane and other intracellular membranes, resulting in plasma membrane recruitment, membrane destabilization, and cell death. The process of destabilizing the plasma membrane, forming pores, and determining the makeup of these pores based on active MLKL (phosphorylated or oligomerized) is still under investigation. The current estimated diameter of the necroptotic pore is approximately 4 nm; however, this size appears insufficient to permit the release of certain inflammatory mediators with molecular weights of ~100 kDa [32]. This suggests that membrane stress generated by small pore formation may trigger secondary, larger membrane ruptures in cells undergoing necroptosis. Moreover, MLKL has not been demonstrated to directly form pores; rather, MLKL oligomerization is thought to destabilize the plasma membrane, thereby promoting permeabilization. Consequently, further investigation into the sequential stages of necroptosis, particularly with respect to pore size dynamics, MLKL activation state, and MLKL localization within the membrane and pore structures, is necessary to fully resolve these outstanding questions.

## 3. Molecular Mechanisms of MLKL in Necroptosis

### 3.1. TNFR1 Signalling Pathway

**Figure 2:**
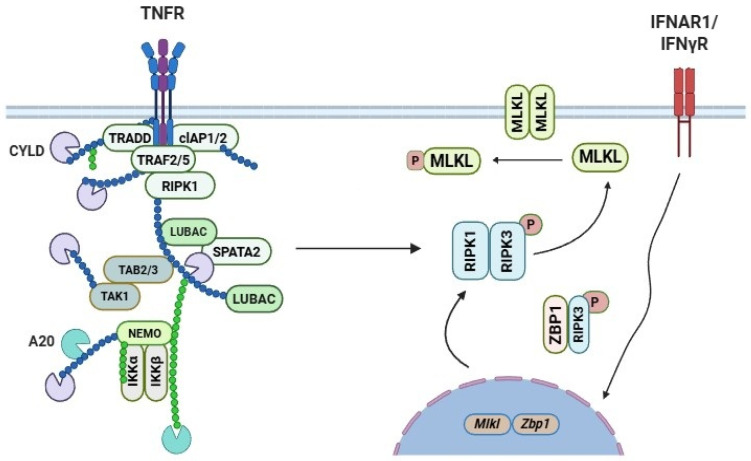
Binding of TNF to TNFR1 promotes assembly of membrane-associated Complex I containing TRADD, TRAF2, cIAP1/2, LUBAC, RIPK1 and IKK1/2, together with mixed polyubiquitin chains, leading to NF-κB and MAPK activation and transcription of pro-survival and pro-inflammatory genes. Interferon signaling induces transcriptional upregulation of ZBP1 and MLKL. ZBP1 senses dsRNA and interacts with RIPK3 via RHIM domains, triggering RIPK3 phosphorylation and MLKL activation. Activated MLKL undergoes a conformational change exposing its N-terminal four-helical bundle (4HB) domain, oligomerizes at the plasma membrane and disrupts membrane integrity, resulting in necroptosis.

Immune receptors can induce necroptosis upon activation by their respective ligands [33]. Among these, the TNFR1-TNF signaling axis is one of the most extensively characterized receptors-ligand systems involved in the initiation of necroptosis [34]. Upon TNF binding to TNFR1, a membrane-associated signaling platform known as complex I is formed [35]. This complex comprises adaptor proteins (e.g., TRADD and TRAF2), E3 ubiquitin ligases (e.g., cIAP1/2 and the linear ubiquitin chain assembly complex, LUBAC), and kinases (e.g., RIPK1 and IKK1/2) (Figure 2) [36,37,38,39]. Complex I promotes activation of the IKK1/2 kinases, leading to NF-κB activation via phosphorylation-dependent degradation of IκBα [40]. A secondary cytoplasmic signaling complex, termed complex II, forms when cIAP1/2 are depleted, LUBAC components (HOIP, HOIL-1, and SHARPIN) are genetically deleted, or IKK activity is inhibited [38,41,42,43,44]. This complex, which includes FADD, Capsase-8, cFLIP, RIPK1, and RIPK3, can cause apoptosis [35,45,46]. TNF causes RIPK1/RIPK3/MLKL-mediated necroptosis when Caspase-8 is inhibited whether pharmacologically, virus-mediated, or genetically [47,48,49,50,51,52,53,54]. Notably, necroptosis can be induced in the absence of RIPK1 [55,56]. These studies identified RIPK1 as a critical homeostatic regulator of cell death, restraining both necroptosis and apoptosis in vivo. In the absence of RIPK1, TRADD is recruited to FADD via death effector domain (DED) interactions, leading to caspase-8 activation. In parallel, RIPK1-independent necroptosis can be initiated through RHIM-mediated interactions between ZBP1 and RIPK3, resulting in MLKL activation [57,58,59].

### 3.2. TLRs Signalling Pathway

TNFR1 is not the only immune receptor capable of inducing necroptosis [33]. Toll-like receptors (TLRs) are pattern recognition receptors (PRRs) that recognize conserved pathogen-associated molecular patterns as well as DAMPs released by injured cells [60,61]. Notably, TLR3 and TLR4 have been shown to induce necroptosis independently of TNF signaling [62]. TLR3 is localized to endosomal compartments and is activated by double-stranded RNA (dsRNA), a viral replication intermediate [63]. Upon activation, TLR3 recruits the adaptor protein Toll/Interleukin-1 receptor domain-containing adapter-inducing interferon-β (TRIF). TRIF plays a dual role in innate immune signaling. Through its pLxIS motif, TRIF engages the kinase TBK1 (TANK-binding kinase 1), leading to phosphorylation and activation of IRF3 and the subsequent induction of type I IFN genes, which are critical for antiviral responses [64]. In addition, TRIF can recruit RIPK3 via RHIM–RHIM interactions, thereby linking pathogen recognition to the activation of necroptosis. This bifunctional capacity allows TRIF to coordinate both antivirals signaling and programmed necrotic cell death, depending on the context of infection or cellular stress. Under conditions of caspase-8 inhibition, TLR3 activation promotes MLKL-dependent necroptosis [11].

While both RIPK1 and RIPK3 are required for MLKL activation in macrophages, RIPK1 is dispensable in fibroblasts and endothelial cells [11]. The mechanism by which caspase-8 is brought into proximity with RIPK3 in the absence of RIPK1 remains unclear, particularly given that RIPK3 lacks both a death domain (DD) and a death effector domain (DED). In contrast, RIPK1 can recruit caspase-8 through DD-mediated interactions with FADD, facilitating assembly of the necroptotic signaling complex. Upon binding lipopolysaccharide (LPS), TLR4 dimerizes and recruits the adaptor protein TIRAP. MyD88 subsequently promotes NF-κB signaling by recruiting TRAF6 to form the Myddosome complex [65]. In parallel, TLR4 can also engage TRIF, thereby recruiting RIPKs and initiating alternative downstream signaling pathways [11,62]. RIPK1 is required for LPS-induced, MLKL-dependent necroptosis in macrophages under conditions of caspase-8 inhibition, but is dispensable in fibroblasts and endothelial cells.

Importantly, combined depletion of cellular inhibitor of apoptosis proteins (cIAP1/2) and inhibition of X-linked inhibitor of apoptosis protein (XIAP), together with LPS stimulation and pharmacological inhibition of caspase-8, allows macrophages to activate MLKL in an RIPK1-independent but RIPK3-dependent manner. This non-canonical necroptotic signaling cascade results in the activation of the NLRP3 inflammasome and subsequent production of IL-1β, likely as a consequence of MLKL-mediated plasma membrane perturbation during necroptotic cell death. These findings highlight a critical crosstalk between necroptotic machinery and innate immune responses: while MLKL executes membrane disruption, RIPK3 serves as the upstream kinase linking apoptotic blockade and pathogen sensing to inflammasome activation. Collectively, these studies demonstrate that IAPs not only inhibit apoptosis but also limit RIPK3-dependent inflammatory cytokine production, revealing a finely tuned balance between cell death pathways and immune activation in macrophages [66,67,68,69]. Collectively, these findings demonstrate that multiple immunological signaling pathways can converge on MLKL activation when caspase-8 function is compromised. MLKL-driven membrane disruption and necroptotic cell death not only eliminate affected cells but also promote inflammasome activation, with the potential to influence neighbouring bystander cells [68,69].

The ripoptosome–necrosome axis plays an important role in signaling downstream of the Fas ligand pathway and TNF-related apoptosis-inducing ligand (TRAIL), both of which converge on the activation of RIPK1 [70,71,72]. Upon ligand binding, the corresponding death receptors undergo trimerization at the plasma membrane, leading to the assembly of the Death-Inducing Signaling Complex (DISC). The DISC core primarily consists of FADD and caspase-8, although additional adaptor and signaling molecules such as TRADD and RIPK1 may also be recruited [73].

Recently, several unexpected regulators of RIPK3 and MLKL-driven necroptosis have been identified that do not depend on the experimental manipulation of caspase-8 activity. These include prolonged hypoxia, which inhibits the oxygen-dependent prolyl hydroxylation of RIPK1, thereby promoting RIPK1-mediated cell death; heat stress, which activates a necroptotic signaling pathway involving ZBP1, RIPK3, and MLKL; and increased intracellular pH resulting from osmotic stress and the activation of the proton efflux pump SLC9A1, which leads to MLKL-driven cell death [74,75,76,77]. These findings, along with studies using mouse models of TNF-driven systemic inflammatory response syndrome (SIRS) and kidney ischemia–reperfusion injury, where loss of MLKL is protective alongside evidence from infectious settings, such as Clostridium or Staphylococcus infections, where MLKL deficiency worsens disease severity, suggest that manipulating necroptotic pathways may have therapeutic benefits in these contexts [78,79,80].

Following DISC formation, RIPK1 is recruited and becomes activated within the complex. If caspase-8 is enzymatically active, the apoptotic cascade proceeds [81]. However, in the absence or inhibition of caspase-8 activity, RIPK1 interacts with RIPK3 to form the necrosome, thereby initiating necroptosis. Similarly, interferons (IFNs) promote the incorporation of RIPK1 into the necrosome through activation of the Janus kinase/signal transducer and activator of transcription (JAK/STAT) signaling pathway. Upon IFN stimulation, JAK/STAT signaling induces transcription of the RNA-activated protein kinase (PKR) gene. The upregulated PKR subsequently interacts with RIPK1, facilitating necrosome assembly and promoting necroptotic signaling [82].

RIPK3 activity is also regulated by dephosphorylation mechanisms. Protein phosphatase 1B (Ppm1b) functions independently of the NF-κB pathway and suppresses TNF-induced necroptosis in quiescent cells, as well as RIPK3 auto-activation. Mechanistically, Ppm1b is thought to target autophosphorylation sites on RIPK3, particularly threonine 231 (T231) and serine 232 (S232), which are critical for its activation [83].

A growing body of research also points to the involvement of acetylation in the control of programmed necrosis, where SIRT1/2 is essential for regulating RIPK1 deacetylation [84,85]. After necrotic stimulation, RIPK1 takes on an active conformation that permits SIRT2, which is present in the pre-formed complex with RIPK3, to deacetylate lysine 530, which is close to RHIM [84].

The recently identified RIPK1–HAT1–SIRT1/2 complex further underscores the critical biological role of deacetylation in regulating cell death pathways. The discovery of multiple acetylation sites on RIPK1 including K115 within the kinase domain and K625, K627, K642, and K648 within the death domain, suggests that acetylation may serve as an important regulatory mechanism in programmed cell death (PCD) [85]. Pharmacological inhibition using a novel pan-sirtuin (SIRT) inhibitor led to increased acetylation of additional lysine residues within the RIPK1 death domain, specifically K596 and K599. This enhanced acetylation further modulated cell death outcomes, highlighting the fine-tuning role of RIPK1 acetylation in controlling cell death signaling pathways [85].

### 3.3. Ubiquitination of MLKL

The ubiquitin system plays a critical role in the regulation of necroptotic signaling [86]. Multiple lysine residues on RIPK1 have been identified as ubiquitin acceptor sites, and ubiquitination of RIPK1 can either promote or suppress necroptosis. Ubiquitination at K376 [86,87] or K634 [86] inhibits RIPK1 kinase activity and suppresses necroptosis [88], whereas ubiquitin conjugation at K115 [89] or K627 [90] enhances RIPK1 kinase activity and promotes necroptotic signaling. In addition, RIPK3 is also regulated by ubiquitination during necroptosis. MLKL is ubiquitinated in the LPS signaling pathway [67], although the role of ubiquitin in controlling MLKL killing capacity is not yet clear.

The first report describing MLKL ubiquitination was published by P. Meier’s group [91]. They demonstrated that MLKL undergoes time-dependent ubiquitination during necroptosis, with the earliest ubiquitin modifications coinciding with MLKL phosphorylation and the initiation of necroptotic signaling. Importantly, pharmacological inhibition of RIPK1 or RIPK3 kinases abolishes these ubiquitin modifications, indicating that MLKL ubiquitination depends on the activity of both kinases. The authors further discovered that K63-linked polyubiquitin chains represent the predominant ubiquitin linkage type on MLKL during necroptosis. Through reconstitution of *Mlkl^−/−^* mouse cells with single or multiple lysine-mutant MLKL variants, the authors demonstrated that mutation of lysine 219 (K219; corresponding to K230 in human MLKL) markedly impaired MLKL killing activity. According to their proposed model, phosphorylation-induced activation of MLKL enables ubiquitin conjugation at K219, which consolidates the active MLKL conformation and augments its killing potential.

In the same year, J. Silke’s group published a study examining the role of MLKL ubiquitination in necroptosis, reporting findings that contrasted with those of Meier and colleagues [92]. Their data indicated that MLKL ubiquitination acts as a negative regulator of necroptosis. Specifically, MLKL was found to be ubiquitinated in the crude membrane fraction following oligomerization and to undergo multi-mono-ubiquitination rather than K63-linked polyubiquitination. Using an N-terminal Flag-tagged MLKL construct that could be phosphorylated, ubiquitinated, and translocated to the membrane without inducing cell death, the researchers observed that MLKL levels at the membrane decreased over time. Treatment with lysosomal and proteasomal inhibitors delayed this time-dependent reduction, suggesting that ubiquitination facilitates MLKL degradation through the lysosome, the proteasome, or both pathways.

Wallach’s group reported a distinct perspective on the biological function of MLKL ubiquitination [93], suggesting a cell death-independent role. Consistent with previous studies, they observed that MLKL undergoes ubiquitination in both human and mouse cells following necroptosis-inducing stimuli. These ubiquitin modifications required MLKL phosphorylation and oligomerization and were predominantly K63-linked. Mass spectrometry analysis identified multiple ubiquitinated lysine residues, with K50 in human MLKL and K50/K51 in mouse MLKL being the most frequently modified. Collectively, three research groups have proposed distinct models for the role of MLKL ubiquitination, highlighting the complexity of this modification. One model suggests that ubiquitination enhances MLKL-mediated cell death, whereas a second proposes that ubiquitination inhibits necroptosis. A third model indicates that MLKL ubiquitination can function independently of cell death, for example, by promoting bacterial clearance.

MLKL appears to be ubiquitinated in both the cytoplasm and at the plasma membrane, with the type of ubiquitin linkage and functional outcome varying by location. For example, cytoplasmic ubiquitination may promote MLKL-mediated cell death, whereas membrane-associated ubiquitination could inhibit it. Distinct subcellular pools of MLKL may therefore be differentially modified, modulating the extent and timing of necroptosis. However, studies using a USP21 fusion construct indicate that, regardless of the specific ubiquitin type, ubiquitination generally restrains necroptosis execution. Notably, under strong necroptotic stimuli, ubiquitinated MLKL can rapidly drive cell death, and ubiquitination at K219 specifically contributes to MLKL oligomerization and cytotoxic activity.

### 3.4. Role of MLKL in Viral Infections

Necroptosis is thought to have originated as an innate immune response to pathogens and block apoptosis in various animals [94]. Pathogens have evolved to avoid necroptosis due to host cells’ ability to trigger it and limit viral multiplication [95]. Viruses can counteract necroptosis by hijacking host cellular machinery to support their replication [94]. Some viruses induce necroptosis to facilitate viral release during the MLKL-driven cellular burst, while others modulate necroptosis to prevent premature cell death, instead exiting the host cell via exosome-like vesicles at the plasma membrane [10]. MLKL contributes to the host response against viral infection through two main mechanisms. In the first, viruses trigger RIPK3-mediated MLKL activation, as observed with Vaccinia virus, Cytomegalovirus (CMV), and Influenza virus. In the second, certain viruses directly target MLKL or its upstream regulators to inhibit necroptosis, including BeAn 58058 poxvirus, Cotia poxvirus, and human CMV UL36 [20,96]. The factors determining why some viruses exploit one mechanism over the other or both simultaneously remain unclear. Research on necroptosis in viral infection has historically focused on RIPK3 activation. This emphasis reflects both the earlier lack of MLKL knockout models and the limited initial recognition of MLKL’s essential role in necroptosis. Unlike *Ripk3*, *Mlkl* mRNA is typically upregulated during inflammatory responses, particularly in the context of type I and type II interferon signaling, which is often triggered during pathogen infection [97]. Activation of transcription factors such as STAT1, STAT2, and IRF9 via IFNAR or type I interferon signaling, as well as cGAS/STING-mediated activation of STAT1, has been shown to upregulate MLKL expression at both the transcriptional and translational levels [26]. In contrast, *Ripk3* mRNA, but not *Mlkl*, is elevated following infections with pathogens such as *Mycobacterium tuberculosis* and *Clostridium difficile*. This upregulation occurs independently of interferon signaling and is driven by promoter demethylation processes [97,98].

The best-known example of virus-induced necroptosis is Vaccinia virus. During infection, virus stimulates TNF production, which activates RIPK3 and subsequently triggers MLKL-mediated necrotic cell death. Mice lacking RIPK3 are highly susceptible to infection, whereas wild-type mice are able to mount an effective immune response, partly through necroptosis-induced inflammation [48]. In contrast, other viruses, including mouse CMVs, BeAn 58058 poxvirus (BAV), Cotia poxvirus (COTV), and human herpes simplex viruses 1 and 2, have evolved mechanisms to inhibit necroptosis, allowing them to evade host defences [20,96,99,100,101,102,103].

Influenza A viruses (IAVs) appear to inhibit cIAP2, leading to increased accumulation of RIPK3 [104]. However, in the absence of RIPK3, viral infection and replication remain unaffected. Following infection, activation of RIPK3 promotes the recruitment of FADD, RIPK1, and MLKL, triggering both MLKL-mediated necroptosis and RIPK3/caspase-8–dependent apoptosis [105]. Specifically, RIPK3 activates RIPK1 and FADD, which facilitates caspase-8 recruitment and induces apoptosis. Deficiency of MLKL shifts cell death toward caspase-dependent apoptosis, whereas loss of RIPK3 inhibits both apoptotic and necroptotic pathways. Recent studies indicate that the DNA sensor ZBP1/DAI initiates RIPK3-dependent signaling in influenza A virus (IAV)-infected cells, promoting necrosome formation and activation of MLKL and caspase-8 [106]. In addition to apoptosis and necroptosis, ZBP1 has also been shown to induce pyroptosis following IAV infection, suggesting that it functions as an apical sensor of IAV-induced cell death.

Cells lacking ZBP1 display greater resistance to virus-induced cell death than RIPK3-deficient cells, underscoring the upstream role of ZBP1. In the absence of RIPK3, ZBP1 can directly interact with RIPK1 to trigger apoptosis through the FADD–RIPK1–caspase-8 axis. Conversely, interaction between ZBP1 and RIPK3 promotes necrosome assembly and subsequent MLKL activation, leading to necroptosis. Loss of RIPK3 or ZBP1, particularly when combined with a noncleavable form of caspase-8, permits uncontrolled IAV replication by disabling host antiviral defences and blocking both apoptotic and necroptotic pathways [105,106,107]. In contrast, MLKL deficiency does not markedly alter susceptibility to IAV infection, likely because apoptosis remains intact through the ZBP1–caspase-8 signaling axis. Consistent with this, mice lacking both RIPK3 and FADD exhibit heightened susceptibility to IAV-induced mortality, as infected cells are unable to undergo programmed cell death and therefore fail to initiate effective antiviral responses [108].

Recent studies indicate that direct targeting of MLKL can suppress necroptosis. Certain poxviruses, including BAV and COTV, encode truncated viral MLKL homologues. These viral MLKL proteins contain the pseudokinase domain (PSKD) but lack the 4HBD). By acting as alternative substrates for RIPK3-mediated phosphorylation, viral MLKL homologues divert RIPK3 away from host MLKL. Consequently, host MLKL remains inactive, preventing execution of necroptosis [20].

In contrast to murine CMV, human CMV inhibits necroptosis without encoding a vIRA-like protein. Expression of the immediate early protein IE1 reduces cell death downstream of MLKL phosphorylation by preventing membrane permeabilization [109]. In addition, the human CMV protein UL36 binds both murine and human MLKL; however, it selectively induces degradation of human MLKL, thereby inhibiting necroptosis in infected human cells [20]. These observations highlight that the virus can directly target MLKL itself, an intriguing strategy given the more widely recognized viral mechanisms that interfere with upstream RIP kinase signaling. MLKL may function not merely as a necroptotic executioner but as a multifunctional antiviral effector and signaling hub. Viral evolution of anti-MLKL mechanisms strongly suggests that its role in infection extends beyond cell death, making MLKL a compelling target for future research into antiviral immunity and viral pathogenesis.

### 3.5. Role of MLKL in Human Chronic Diseases

MLKL has emerged as a significant contributor to the pathogenesis and progression of numerous human chronic diseases beyond its role in acute cell death. Dysregulated MLKL activation and necroptotic signaling are implicated in chronic inflammatory, cardiovascular, metabolic, neurodegenerative, fibrotic, and cancerous conditions, where persistent necroptosis and release of DAMPs drive sustained inflammation and tissue injury rather than protective cell clearance. In humans with unstable carotid atherosclerosis, expression of RIP3 and MLKL is increased, and MLKL phosphorylation, a key step in the commitment to necroptosis, is detected in advanced atheromas [110] and correlates with markers of inflammation and insulin resistance, suggesting that MLKL contributes to lesion progression and cardiovascular risk, particularly in patients with type 2 diabetes mellitus [111]. Using a radiolabeled necroptosis inhibitor, ^123^I-Nec-1, necroptotic activity was selectively detected within atherosclerotic plaques of Apoe^−/−^ mice, with tracer uptake closely correlating with lesion size. Moreover, pharmacological inhibition of necroptosis with Nec-1 in mice with established atherosclerosis reduced plaque burden and features of plaque vulnerability, including necrotic core formation, thus supporting that targeting necroptosis may represent a promising therapeutic strategy to slow or prevent atherogenesis. MLKL appears to regulate endothelial and macrophage responses to oxidized lipids, promoting pyroptotic and inflammatory signaling pathways that exacerbate vascular inflammation [110,111,112]. Similarly, chronic activation of MLKL-mediated necroptosis also drives cardiac injury in immune-associated myocarditis and diabetic cardiomyopathy, where pharmacological inhibition or genetic deletion of MLKL attenuates cardiomyocyte death, fibrosis, and functional decline [113,114]. In liver disease, MLKL-dependent necroptosis fosters chronic inflammation and fibrogenesis, linking necroptotic signaling with progression from steatosis to fibrosis and eventual tumorigenesis in experimental models, and MLKL participates in hepatic stellate cell activation and inflammasome assembly in chronic liver injury [115]. Beyond cardiometabolic and fibrotic disease, MLKL influences neurodegenerative processes, where its deficiency reduces neuroinflammation and neuronal loss in models of Parkinson’s disease, and chronic sterile inflammation associated with aging is ameliorated in *Mlkl*-deficient mice [116,117]. In cancer, MLKL expression and activation show complex, context-dependent associations with tumor progression and patient outcomes [118], reflecting necroptosis-dependent and necroptosis-independent functions that influence inflammatory microenvironments, cell trafficking, and tumor cell survival [119]. Collectively, these observations underscore that MLKL acts not only as an executioner of necroptosis but also as a modulator of chronic inflammation, tissue remodeling, and disease progression, making it a compelling focus of translational research and therapeutic targeting in human chronic diseases.

## 4. Therapeutic Mechanisms Targeting MLKL

Therapeutic targeting of regulatory checkpoints involved in MLKL activation has the potential to prevent and treat associated disorders, as MLKL is a critical mediator of necroptosis and inflammatory signalling.

### 4.1. Targeting MLKL Oligomerization, Membrane Binding, and Insertion

The formation of MLKL oligomers largely depends on interactions between the N-terminal execution domain (NED) and the brace region. The first known MLKL inhibitor, Cpd 7 (NSA), is a small molecule that covalently binds to the NEDH4 domain at Cys86 via a Michael acceptor [28]. Subsequent studies demonstrated that NSA inhibits the oligomerization of MLKL tetramers, thereby preventing necroptotic cell death. Furthermore, NSA does not completely block MLKL tetramer formation [119]. It has been shown to cross-link Cys86 of human MLKL with Cys32 of Trx1, thereby preventing disulfide bond formation and MLKL polymerization in vitro [120]. In addition, NSA can suppress both necroptosis and pyroptosis by covalently targeting critical cysteine residues (Cys191/Cys192) on GSDMD [121]. Following the discovery of NSA, which established the potential of targeting C86 to inhibit necroptosis, Wang et al. identified Cpd 8, a xanthine-based covalent inhibitor of C86, through high-throughput screening [122]. In parallel, the classical xanthine inhibitor Cpd 10 (BI-8925) was also found to suppress necroptosis in Jurkat and U937 cells, highlighting the broader applicability of xanthine scaffolds in necroptosis modulation.

Cui et al. optimized TC13172 to develop highly potent compounds 11 and 12, featuring uracil-based core structures [123]. These compounds inhibited MLKL oligomerization while markedly reducing membrane translocation, thereby minimizing off-target effects and cytotoxicity [123]. These findings suggest that C86 does not directly regulate necroptosis. However, the C86 covalent inhibitor in H4 cells appears to mimic the effect of S83 phosphorylation [124]. Rübbelke et al. [125] employed protein-detection NMR to identify Cpd 13, a non-covalent MLKL NED inhibitor. Through scaffold hopping, they developed the affinity-enhanced Cpd 14, featuring an iso-propylphenyl group positioned in the hydrophobic core near H2 and H5. NMR relaxation studies and X-ray crystallography revealed that Cpd 14 adopts a flexible, partially open conformation induced by the H2–H3 loop. While these compounds can inhibit the binding of monomeric detergent molecules, they do not effectively stabilize the 4HB domain or the auto-inhibitory interaction of H6, and thus fail to prevent oligomerization [125].

BMS-777607, a TAM kinase inhibitor, regulates MLKL oligomerization without affecting phosphorylation or membrane translocation [31]. NBC1, an HSP70 inhibitor, disrupts the interaction between the SBD and MLKL NED, promoting MLKL polymerization and stability but not tetramerization [126,127]. AMG-47a, a lymphocyte-specific Lck inhibitor, interacts with RIPK1 and RIPK3, acting on a step downstream of MLKL dimerization and potentially impeding MLKL oligomerization or membrane translocation [16,128]. Additionally, Pierotti et al. identified ABT-869 (Linifanib), a VEGFR/PDGFR tyrosine kinase inhibitor that targets RIPK1 and inhibits cell death in cells expressing self-activated MLKL mutants [129].

### 4.2. Targeting Pseudokinase Domain of MLKL

Although MLKL lacks catalytic kinase activity, its pseudokinase domain can bind ATP and be activated by RIPK3 [14,130]. Using a thermal shift assay, Hildebrand et al. screened 367 small molecules against the recombinant mouse MLKL pseudokinase domain and identified Cpd 1 (an ATP analogue, also known as GW806742X) as a binder of MLKL [16]. Surface plasmon resonance (SPR) analysis confirmed that GW806742X binds the MLKL pseudokinase domain [16]. Although it did not inhibit RIPK3-mediated phosphorylation, GW806742X protected MDFs from TNF/Smac/Q-VD-OPh-induced necroptosis at an EC of 1.85 μM, but lost efficacy at concentrations above ~2 μM [131]. The compound was also associated with hepatotoxicity and gastrointestinal adverse effects, and its anti-necroptotic activity may stem from nonspecific binding to RIPK1 rather than selective MLKL inhibition [131]. The anti-necroptotic activity of nucleotide analogues cannot be fully assessed until MLKL-specific effects are confirmed. To address this, the authors developed Cpd 4, an MLKL-selective analogue, along with two truncated derivatives, Cpd 2 and Cpd 3, which exhibit improved selectivity against RIPK1. However, none of these compounds rescued cells from necroptosis. Similarly, the MLKL-binding agent Cpd 5 (Crizotinib) also failed to confer protection against necroptotic cell death [131].

Prajapati et al. [132] reported that Cpd 6 (6,7-Dihydroxycoumarin, 6,7-DHC) exhibits both preventive and therapeutic effects in oxalic acid-induced chronic kidney disease mouse models. Using an unbiased high-content in vitro screening approach, the authors found that 6,7-DHC interacts with MLKL and suppresses its phosphorylation. However, the compound also reduced RIPK1 and RIPK3 phosphorylation, suggesting a broader mechanism of action beyond MLKL inhibition [133]. To date, studies with MLKL pseudokinase domain binders have generally demonstrated limited efficacy in modulating the necroptotic pathway. Appropriate ATP analogues targeting the MLKL pseudokinase domain may interfere with its switching mechanism, inducing conformational changes that limit access to the activation loop and inhibit phosphorylation [131].

### 4.3. Targeting MLKL Translocation to the Plasma Membrane and Membrane Binding

During the effector stage of necroptosis, MLKL is trafficked to the plasma membrane through Golgi–microtubule–actin-dependent processes. Treatment with a combination of Nocodazole, Cytochalasin B, and Brefeldin A (NCB) effectively prevents PM disruption at any stage of necroptosis. While NCB delays MLKL membrane binding, it does not completely block it, resulting in a smaller “hot spot” of membrane-associated MLKL [133].

Petrie et al. [134] developed monobodies (synthetic binding proteins, Mb33 and Mb37) to investigate regulatory checkpoints during necroptosis. These monobodies bind the H6 helix and adjacent loop of human MLKL [134]. While they are unlikely to block MLKL tetramerization, they may impede the release of the 4HB domain from the brace region, thereby inhibiting membrane binding [135]. Similarly, Liu et al. reported that repulsive guidance molecule b (RGMb) reduces MLKL membrane translocation and binding without affecting phosphorylation or oligomerization, ultimately preventing membrane association and necroptotic cell death [136].

### 4.4. Targeting Stability of MLKL

S-phase kinase-associated protein 2 (Skp2) is an E3 ubiquitin ligase and a key component of the SCF (Skp1–Cullin1–F-box) complex. Zhou et al. [137] demonstrated that Skp2 mediates the ubiquitination and degradation of MLKL [121]. In this context, the Skp2 inhibitor SZL P1-41 was shown to disrupt MLKL binding and prevent its degradation in A549 cells [137]. Rathje et al. applied the PROteolysis TArgeting Chimera (PROTAC) strategy to degrade MLKL via the E3 ubiquitin ligase cereblon (CRBN), in addition to Skp2. These PROTACs combined a high-affinity pyrazole carboxyamide MLKL ligand with lenalidomide as the CRBN-recruiting moiety. Among them, Cpd 15 emerged as the most potent MLKL degrader, achieving near-complete degradation in a dose-dependent manner. However, its efficacy in vivo models have not yet been evaluated [138].

Li et al. [139] developed covalent PROTACs by optimizing a theophylline derivative, ultimately identifying Cpd 16 (MP-11) as an effective MLKL degrader. Although Cpd 16 was able to degrade MLKL in mouse xenograft models, its covalent binding to C86 of human MLKL, a residue not conserved in mice, limits the interpretation of its in vivo activity. Consequently, the covalent mechanism constrains the broader applicability of this compound in preclinical studies.

### 4.5. Natural Products Targeting MLKL

Natural products are a diverse class of biologically active substances produced by living organisms, including microorganisms, plants, animals, insects, and marine species [140,141]. Owing to their structural diversity and wide range of biological origins, natural products represent a rich and valuable resource for the discovery of MLKL-related therapeutic agents. Several natural compounds have been identified as regulators of MLKL expression, including ligustroflavone, hesperetin, and calycosin. Ligustroflavone, derived from *Ligustrum lucidum*, has been shown to downregulate MLKL expression and exert potent anti-inflammatory and therapeutic effects in disease models such as ischemic stroke [142,143]. Hesperetin, a flavanone abundantly found in citrus fruits, consistently attenuates inflammation in colitis by suppressing MLKL expression, inhibiting necroptosis, and modulating immune responses [144,145,146]. In addition, the stability of MLKL expression depends on the interaction between Krüppel-like factor 2 (KLF2) and MLKL, an interaction that can be directly disrupted by calycosin, a bioactive isoflavone isolated from *Radix astragali* [147,148].

Several natural compounds have been reported to interfere with MLKL phosphorylation, including tetramethylpyrazine, celastrol, luteolin, and esculetin. Tetramethylpyrazine, derived from *Ligusticum wallichii*, and celastrol, isolated from *Tripterygium wilfordii*, have been shown to prevent cell necrosis and tissue injury by inhibiting MLKL phosphorylation [149,150]. In addition, luteolin, a flavonoid extracted from *Rhizoma Drynariae*, may directly bind to MLKL, thereby preventing its phosphorylation and subsequent execution of necroptosis [151]. By contrast, esculetin [6,7-dihydroxycoumarin (DHC)] appears to inhibit the ATP-binding site of MLKL, leading to reduced MLKL phosphorylation and activity [132]. Baicalin, an antagonist of MLKL oligomerization, is isolated from the roots of *Scutellaria baicalensis* Georgi. Although baicalin does not affect the phosphorylation of RIPK1, RIPK3, or MLKL, it significantly inhibits MLKL oligomerization, thereby suppressing necroptosis and alleviating associated pathological processes such as inflammation [152,153].

## 5. Conclusions

Over the past decade, necroptosis has emerged as a central host defense mechanism against infections, prompting pathogens to evolve multiple strategies to evade or suppress this pathway. Although MLKL is firmly established as the terminal effector of necroptosis, fundamental questions regarding its regulation remain unresolved. In particular, whether MLKL can induce cell death independently of RIPK3, or be activated through alternative phosphorylation events or kinases, remains unclear. Current evidence strongly supports RIPK3-mediated phosphorylation at sites other than S345 and S357/T358 as the dominant mechanism driving TNF-induced necroptosis, yet additional layers of regulation may exist. Beyond its canonical role in necroptosis, accumulating evidence suggests that MLKL may participate in diverse signaling networks, including crosstalk with caspase-dependent cell death pathways, potentially being activated through currently unknown mechanisms. Elucidating how MLKL transitions between inactive, activated, and inhibitory states is therefore critical for delineating its physiological and pathological functions. Despite extensive research, many aspects of MLKL function remain poorly understood. It is unknown whether distinct stimuli induce different MLKL oligomeric assemblies, how the 4HB domain engages and remodels the plasma membrane to initiate pore formation, or how membrane composition and specific lipid interactions influence MLKL oligomerization and cytotoxic activity. Since we lack a universal, high-affinity inhibitor that can target the 4HB-lipid engagement site across species, resolving these questions is crucial for moving therapies from preclinical models to humans. From a translational perspective, advances in diagnostic tools, such as monoclonal antibodies recognizing phosphorylated human MLKL in tissue biopsies, offer promising opportunities to identify and quantify necroptotic cell death in human pathology, analogous to cleaved caspase-3 as a marker of apoptosis. However, species-specific differences between human and murine MLKL continue to limit the direct translation of such tools to preclinical models, underscoring the need for improved reagents and standardized biomarkers of MLKL activation. Therapeutically, the limited number of MLKL-targeted therapies currently in development reflects both the complexity of MLKL biology and the pharmacological challenges associated with its modulation. Future efforts should prioritize the development of potent, selective, bioavailable, and non-toxic MLKL inhibitors and evaluate their utility in disease contexts.

## Data Availability

No new data were created or analyzed in this study.

## Abbreviations

The following abbreviations are used in this manuscript:

MLKL	Mixed lineage kinase domain-like protein
RIPK3	Receptor-interacting protein kinase 3
DAMP	Damage-associated molecular patterns
TNFR1	TNF receptor 1
DAI	DNA activator of interferons
4HB	Four-helix bundle
ZBP1	Z-form RNA binding protein 1
FADD	Fas-associated protein with a death domain
TRIF	TIR-domain-containing adapter-inducing interferon β
PSKD	Pseudokinase domain
TRADD	TNF receptor-associated death domain
LUBAC	Linear ubiquitin chain assembly complex
DED	Death effector domain
RHIM	RIP homotypic interaction motif
TLRs	Toll-like receptors
TBK1	TANK-binding kinase 1
IFN	Interferons
cIAP1/2	Cellular inhibitor of apoptosis proteins
CMV	Cytomegalovirus
IAVs	Influenza A viruses
RGMb	Repulsive guidance molecule b
PROTAC	PROteolysis TArgeting Chimera
KLF2	Krüppel-like factor 2
